# The incidence and epidemiology of necrotising otitis externa in England before, during and after the coronavirus disease 2019 pandemic: an updated analysis of Hospital Episode Statistics data 2002–2024

**DOI:** 10.1017/S0022215125102910

**Published:** 2025-10

**Authors:** Donny Kong, Stefan Linton, Emma Stapleton

**Affiliations:** 1 Department of Otolaryngology, Manchester Royal Infirmary, Manchester, UK; 2 Manchester Academic Health Sciences Centre, University of Manchester, Manchester, UK

**Keywords:** diabetes mellitus, immunosuppression, osteomyelitis, otitis externa

## Abstract

**Objectives:**

Necrotising otitis externa is a serious infective condition with significant risk of complications and a profound impact on patients’ quality of life.

**Methods:**

A quantitative descriptive study was undertaken using epidemiological data from the National Health Service Hospital Episode Statistics database and other national databases. Data correlating with reported cases 2002-2024 were compiled and analysed.

**Results:**

The national incidence of necrotising otitis externa has demonstrated a sustained increase 2002-2024. The 30 per cent incidence drop during the coronavirus disease 2019 pandemic may be attributable to reduced exposure to risk factors, reduced contact between susceptible patients and health professionals and pandemic-related deaths of at-risk populations. There remains a strong correlation between growths in necrotising otitis externa incidence, the ageing population and national incidence of diabetes mellitus. These are all projected to continue to rise. Antibiotic resistance is not a significant contributing factor.

**Conclusion:**

This study demonstrates several significant trends, offering a strong foundation for deeper exploration in future studies.

## Introduction

Necrotising otitis externa (NOE) is a serious infective condition of the external auditory canal, first comprehensively described by Chandler in 1968.^1^ Originally termed “malignant external otitis” the term *necrotising otitis externa* is preferred by both clinicians and patients^2^ as it avoids connotations of neoplasia. The condition is largely but not exclusively diagnosed in frail patients with recognised risk factors including advanced age, diabetes, immunosuppression and epidermal compromise of the external auditory canal skin.^3^

NOE carries a significant risk of serious complications^4^ and has a profound impact on patients’ quality of life.^5^ It frequently requires lengthy treatment protocols, multiple investigations and inpatient care, rendering it a considerable burden to health systems.^6^ However, until recently, heterogeneous diagnostic criteria were applied across published studies,^7^ and the reporting of outcomes has been extremely variable.^8^ This has prevented the synthesis of data and could be mitigated by the implementation of the recently published Core Outcome Set for Necrotizing Otitis Externa (COSNOE) consensus diagnostic criteria and core outcome set for NOE^2^ in the longer term.

NOE has attracted the attention of clinicians and researchers due to its apparent rising incidence in the United Kingdom^9^ which has been correlated with an increasing prevalence of diabetes mellitus, an ageing population and enhanced clinician awareness of NOE.^10^ Antibiotic resistance has been proposed as a contributing factor,^11^^,^^12^ though this is not a universal observation, with other studies reporting a steady incidence of resistant strains of *Pseudomonas aeruginosa* over time.^4^ In contrast to the apparent rising incidence of NOE in England, reported data from other countries includes epidemiological studies from Taiwan where the incidence of NOE has decreased between 2001 and 2015^13^ and the USA where an audit of the National Inpatient Sample Database^14^ demonstrated a steady incidence of NOE between 2002 and 2013.

The most recently published analysis of national NOE data was our publication in the JLO reporting the rise in annual incidence of NOE in England between 2002 and 2018^10^ and exploring theories for this phenomenon. Since then, the Covid-19 pandemic has had a profound impact on otorhinolaryngological disease presentation,^15^ prevalence^16^ and management,^17^ with a plethora of publications describing the impact of national lockdowns and limited access to healthcare during the pandemic,^18^ though many of these are single-centre studies, and therefore do not fully explore the impact of the pandemic on a national level.

No publication has yet analysed the impact of the Covid-19 pandemic on the incidence of NOE. This is surprising, considering the classic demographic of a patient with NOE (elderly, frail, diabetic or immunocompromised) aligns with the at-risk populations advised to shield during the Covid-19 pandemic. The aim of this study was therefore to produce an updated analysis of national Hospital Episode Statistics (HES) data 2002-24; to map the changing incidence of NOE before, during and after the Covid-19 pandemic; and to revisit the epidemiological correlations observed in our previous publication.^10^

## Materials and methods

Data on hospital admissions for NOE in England from 2002 to 2024 were collected from the HES database available through the NHS digital portal.^19^ The dataset encompassed patient demographics, admission counts and hospital stay durations. Relevant data for adult diabetes mellitus types 1 and 2 from 2003 to 2024 were sourced from the National Diabetes Audit and the Quality and Outcomes Framework database.^20^

To assess improved clinician awareness, ENT publication trends in the management of NOE, across ENT journals with an impact factor greater than 1 were reviewed between the period of 2002 and 2024. Two researchers independently conducted exhaustive searches across Medline, Embase, PubMed and the Cochrane Library using relevant keywords. They identified relevant publications from 2002 to 2024 and verified the findings by cross-checking studies and reference lists.

In the absence of a national antibiotic resistance database, ciprofloxacin resistance data for *P. aeruginosa* from adult ear swabs collected between 2002 and 2024 were compiled from the local microbiology department within our tertiary centre. This database including samples from a wide geographical area encompassing both primary and secondary care settings.

## Results

**Figure 1.**
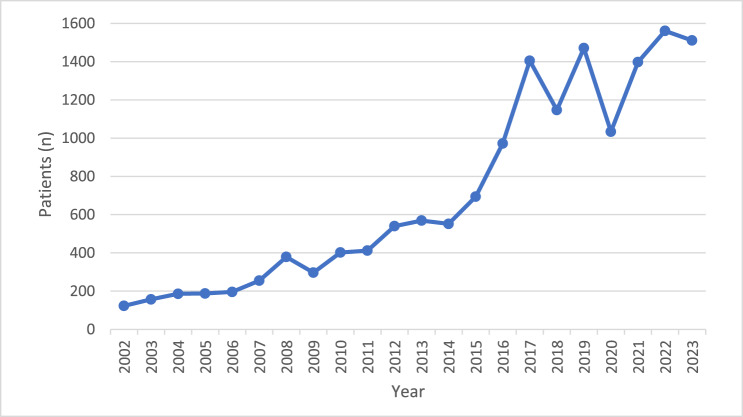
Chart showing rise in NOE cases 2002–2024.

Between 1 April 2002 and 31 March 2024, there were a total of 15449 reported NOE cases accounting for 142,002 hospital bed days. Of these patients, (71.2 per cent) were male and (28.8 per cent) were female. The mean hospital stay was 15.9 days (range 11.7-18.5 days). Admission data (Table 1 and Figure 1) show a gradual increase in reported cases annually from 2002-2003 to 2014-2015, followed by a marked rise between 2014-2015 and 2017-2018, and several peaks and troughs between 2017-2018 and 2023-2024 with an overall continued rise in cases per year. For brevity, years referred to in the charts refer to the period commencing 1 April of that year and ending 31 March the following year.

**Table 1.** Hospital Episode Statistics for NOE

Year	NOE hospital admissions (n)	Mean length of hospital stay (days)	Total bed days for NOE admissions (n)
2002−3	123	11.7	1130
2003−4	157	15.7	1943
2004−5	186	14.6	2076
2005−6	188	15.3	2277
2006−7	196	14.6	2011
2007−8	255	16.3	3021
2008−9	379	18	3879
2009−10	297	15.5	3564
2010−11	402	16.4	4761
2011−12	412	15.9	4959
2012−13	540	16.8	6117
2013−14	569	18.5	6480
2014−15	552	19	7440
2015−16	694	21	8708
2016−17	972	16	9642
2017−18	1,405	16	12303
2018−19	1,147	15	11766
2019−20	1,471	16.2	15346
2020−21	1,034	13	7832
2021−22	1,398	13.6	11513
2022−23	1,561	15.9	15234
2023−24	1,511	15.7	14888

Abbreviation: NOE, necrotising otitis externa.

**Figure 2.**
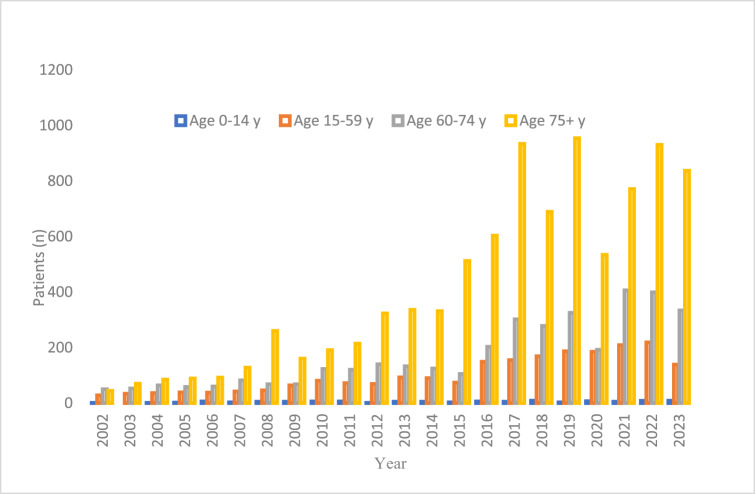
Chart showing changes in NOE patient age 2002–2024.

**Figure 3.**
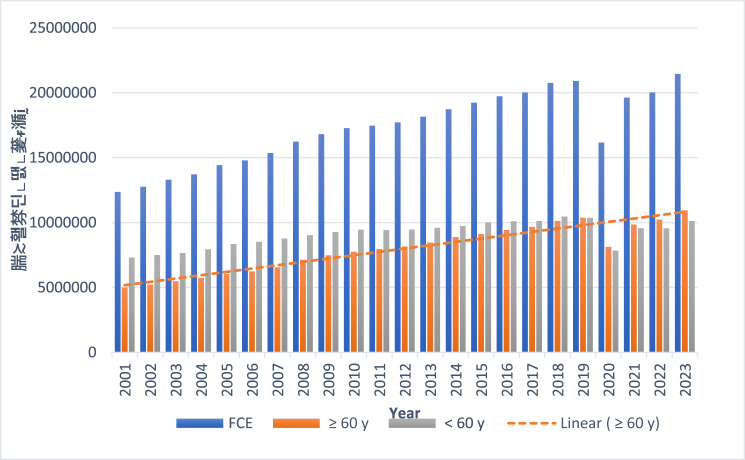
Age trends for all hospital admissions during the same time period.

Most reported cases involved older adults, with 58.8 per cent of patients aged 75 or older and 82.7 per cent aged 60 or older. Over the 22-year span, the average age of patients admitted with NOE steadily rose, with the proportion of patients over 75 increasing from 36 per cent in 2002 to 55.4 per cent in 2023 (Figure 2). For comparison, Figure 3 illustrates the age trends for all hospital admissions during the same period.

**Figure 4.**
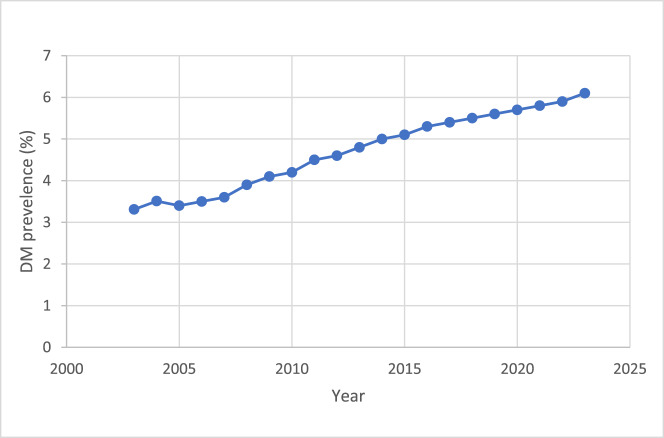
Combined prevalence of diabetes mellitus (DM) types 1 and 2 (data from National Diabetes Audit and Quality and Outcomes Framework UK database).

**Figure 5.**
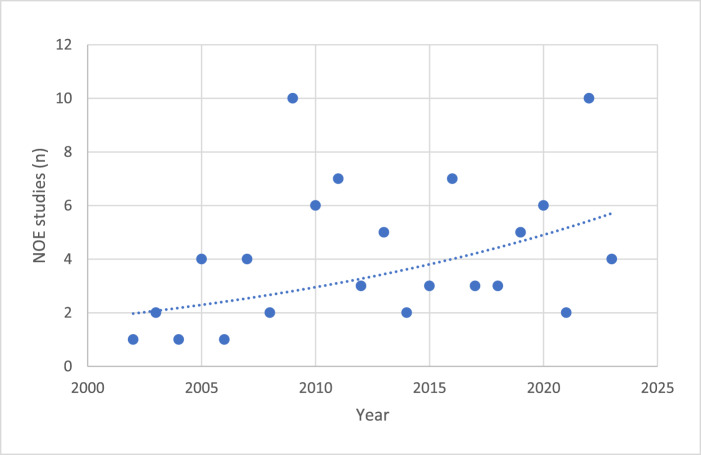
Published peer-reviewed studies of necrotising otitis externa (NOE) management in ENT journals with an impact factor greater than 1 published between 2002 and 2024.

The prevalence of diabetes mellitus types 1 and 2 in England also saw a notable rise, growing from 3.3 per cent in 2003 to 6.1 per cent in 2024 (Figure 4), highlighting a growing population at risk for NOE. The number of peer-reviewed publications on NOE management in ENT journals with an impact factor greater than 1 remains relatively small. However, there was a gradual increase observed over the 22-year period (Figure 5).

**Figure 6.**
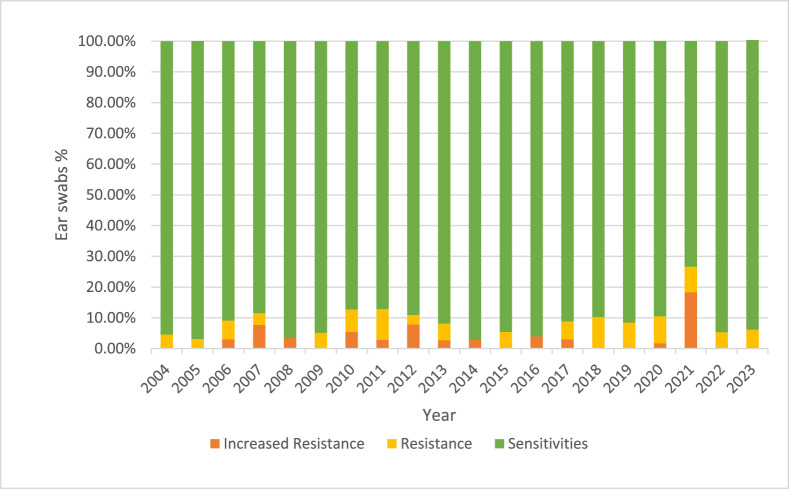
Ciprofloxicin sensitivities for *Pseudomonas aeruginosa* in over 7000 adult ear swabs at Manchester University NHS Foundation Trust between 2002 and 2024.

Analysis of over 7,000 ear swab results from our NHS Trust revealed no significant change in the rate of ciprofloxacin-resistant *P. aeruginosa* over time (Figure 6). Peak resistance levels remained below 10.5 per cent, and the overall proportion of antibiotic-resistant samples remained stable.

It is worth noting that, in 2021, the number of reported resistant cases appeared to increase due to the adoption of new European Committee on Antimicrobial Susceptibility Testing (EUCAST) breakpoint data, which does not reflect a true rise in resistance but rather a change in the criteria for classification.

## Discussion

The increasing incidence of NOE in the UK has been highlighted in multiple publications, with researchers suggesting various potential causes for this trend. Data from the HES reflect a continued rise in the incidence of NOE in England from 2002 to 2024; in contrast to stable or declining rates reported in other countries.^13^^,^^14^

In England, the incidence of NOE rose dramatically between 2002 and 2018, with reported cases subsequently dropping over 18 per cent (from 1405 to 1147 cases per year) between 2017-2018 and 2018-2019, then rising by 28 per cent to 1471 cases per year in 2019-20. This dramatic trough preceded the Covid-19 pandemic by several years; however, the subsequent recovery in incidence suggests an aberration rather than an explicable trend.

The incidence of NOE in England April 2020 – March 2021 represents a second trough, dropping nearly 30 per cent (from 1471 to 1034 reported cases). This trough corresponds with the initial lockdown periods of the Covid-19 pandemic. Although the previous incidence trough might be considered a statistical aberration, the 30 per cent decline in reported NOE cases during the Covid-19 pandemic could be due to several factors, including a reduction in apparent cases due to patients failing to present to hospital during the Covid-19 pandemic or even a decrease in the at-risk population (elderly, frail immunocompromised patients), the same population at risk of death due to Covid-19 virus infection. Alternatively, the decrease in NOE incidence might be a genuine drop in cases due to reduced exposure of at-risk patients to aural irrigation (syringing) or ear canal instrumentation, including other activities such as swimming or holidaying in warm, moist environment, which are well recognised as risk factor for NOE.^3^

After the Covid-19 pandemic, national data demonstrate a continued overall rise in the incidence of NOE, with a 35 per cent increase in incidence in the year following the Covid-19 pandemic (April 2021 – March 2022) and a continued though more modest rise in incidence to April 2024. We revisit four theories to explain this continued apparent rise in the incidence of necrotising otitis externa in England.

Analysis of mortality rates over time in the context of the apparent rising incidence of NOE, would be a useful insight. However, HES data do not incorporate patient-level data or cause-specific mortality data. Although these are theoretically accessible through linking HES data to other datasets such as Office for National Statistics (ONS) mortality data and Civil Registration data, the potential for error is significant, for example, missing data and the documentation of cause of death in frail, multi-morbid patients who are not in hospital. Prospective, large-scale cohort analysis may be useful but would be biased towards the pathways of patients in whom NOE had been correctly diagnosed.

### Rising prevalence of diabetes

Diabetes mellitus, both type 1 and type 2, has long been an associated risk factor for NOE. A population case–control study by Tzong-Hann Yang *et al*.^21^ found that patients with NOE had a significantly high correlation with diabetes (54.8 per cent). As did a study from Stern Shavit *et al*. which showed that 75 per cent of NOE patients had a previous diagnosis of diabetes.^12^ The reported prevalence of pre-existing diabetes among NOE patients in recent studies has noted to vary from 48 to 100 per cent.^7^

Many studies indicate diabetes heightens the risk of NOE due to several inter-related factors. It often leads to impaired blood circulation and ischaemia in the outer ear canal and surrounding tissues, compromising the body’s ability to fight infections. Diabetes weakens the immune response by affecting the function of polymorphonuclear cells and macrophages, impairing their ability to chemotax, phagocytise and kill microorganisms. Increased adherence of pathogens to tissue cells and the enhanced virulence of certain microorganisms in a high-glucose environment further exacerbate the risk. These combined factors create a more conducive environment for severe infections.

The rise in NOE cases parallels an increase in diabetes prevalence, suggesting a potential correlation. The compromised immune response, impaired wound healing and vascular changes associated with diabetes contribute to this heightened risk. Data from the National Diabetes Audit and various epidemiological studies indicate a growing diabetic population, which could partly explain the surge in NOE cases. Whilst the prevalence of diabetes mellitus continues to rise, the trend of rising cases of NOE is unlikely to plateau.

### Ageing population

It is well recognised that most patients with NOE are above the age of 60 years.^10^ Older individuals are more susceptible to NOE for several reasons. Immunosenescence is a decline in immune system function with age, which diminishes the body’s ability to fight infections effectively. This decreased immune response makes it harder for older adults to combat aggressive pathogens, such as *P. aeruginosa*, that commonly cause NOE.

Additionally, older adults are more likely to have underlying health conditions such as diabetes, which further compromises their immune defences and increases their risk of developing severe infections. The natural aging process also contributes to thinning and atrophy of the skin in the ear canal, making it more vulnerable to injury and infection. Furthermore, age-related changes in the ear canal, such as reduced cerumen production and alterations in the microenvironment, can facilitate the growth of pathogenic microorganisms.

Between 2002 and 2024, 58.8 per cent of patients diagnosed with NOE were 75 years or older, and 82.7 per cent were aged 60 years or older. The mean age of patients admitted with NOE consistently increased over the 22-year period, reflecting broader demographic trends.

In England, the population is expected to continue to age, with projections by the Office for National Statistics^22^ suggesting that the proportion of individuals aged 75 and older will continue to increase over the next few decades. As age is a recognised risk factor for NOE, it is therefore likely that the incidence of NOE will continue an upwards trend.

### Increased clinician awareness

The growing incidence of NOE has highlighted the importance of early diagnosis and intervention. A significant factor contributing to the apparent incidence rise may therefore be an increasing awareness of the condition among healthcare professionals, leading to more frequent diagnoses and reporting.

A key factor in raising awareness is the role of continuing medical education (CME) programs and the dissemination of updated clinical guidelines. Improvements in national reporting systems and databases have allowed for more accurate tracking of NOE incidence. The HES database, which compiles data on hospital admissions in England, has become a critical tool for understanding NOE trends. As physicians become more aware of the importance of reporting NOE cases, the number of recorded instances naturally rises.

We used peer-reviewed publications as a proxy to assess whether physician awareness of NOE has increased. However, this approach has significant flaws. While the number of published studies per year showed a gradual increase, the overall volume was too small to draw any definitive conclusions.

### Antibiotic resistance trends

*P. aeruginosa* is the most common bacteria cultured from the affected ears of NOE patients. Given the rise in antibiotic resistance among many bacterial pathogens, the absence of an increase in resistance among *P. aeruginosa* strains associated with NOE is surprising.

Our results highlight that, despite global trends of rising antibiotic resistance, particularly among gram-negative organisms like *P. aeruginosa*, the isolates from NOE cases in our study population showed consistent susceptibility patterns to key antipseudomonal antibiotics. This stability in antibiotic susceptibility is encouraging and suggests that current empirical treatment regimens for NOE remain effective. The continued susceptibility may be attributed to several factors, including the targeted nature of antimicrobial therapy for NOE, which typically involves early recognition of the pathogen and rapid initiation of potent antipseudomonal agents, limiting the time for the development of resistance.

Ciprofloxacin, a fluoroquinolone antibiotic, acts against *P. aeruginosa* by inhibiting two key bacterial enzymes involved in DNA replication: DNA gyrase (topoisomerase II) and topoisomerase IV. By binding to these enzymes, ciprofloxacin stabilises the DNA-enzyme complex, causing breaks in the bacterial DNA and preventing replication and transcription. This disruption of DNA replication and cell division leads to bacterial cell death. Ciprofloxacin’s ability to penetrate bacterial cells and its broad spectrum of activity make it particularly effective against gram-negative organisms like *P. aeruginosa*.

Whilst our findings are reassuring, it is essential to recognise that *P. aeruginosa* remains a pathogen with significant adaptive capabilities. There is still potential for resistance to emerge, particularly given the organism’s ability to acquire resistance genes through horizontal gene transfer and its inherent mechanisms, such as efflux pumps and porin channel modifications. Continuous surveillance of susceptibility patterns in NOE is crucial to ensuring that treatment remains effective over time.

- Necrotising otitis externa (NOE) is a serious infective condition which carries a significant risk of complications and has a profound impact on patients’ quality of life.
- This is the first study to analyse changes in national incidence of NOE before, during and after the coronavirus disease 2019 (Covid-19) pandemic, and projecting likely trends in its future incidence.
- The 30 per cent drop in national NOE incidence during the Covid-19 pandemic may have been due to reduced patient exposure to risk factors, reduced contact between susceptible patients and health professionals or a relative drop due to death rates among at-risk populations.
- There is a strong correlation between the incidence of NOE, the ageing population and national incidence of diabetes mellitus types 1 and 2. These are all projected to continue to rise.
- Antibiotic resistance does not appear to be a significant contributing factor.
- While this study has limitations, its findings point to several significant trends, offering a foundation for deeper exploration in future studies.

## Conclusion

This is the first study to analyse changes in national incidence of NOE before, during and after the Covid-19 pandemic, using large-scale data to explain these changes, and projecting likely trends in its future incidence.

The 30 per cent drop in NOE incidence during the Covid-19 pandemic may have been a real drop due to reduced exposure to risk factors, an apparent drop due to reduced contact between susceptible patients and health professionals or a relative drop due to catastrophic death rates among at-risk (elderly, frail and immunocompromised) patients due to the Covid-19 virus.

Our analyses reveal a strong correlation between the incidence of NOE and a rise in susceptible populations due to corresponding rises in the ageing population and rising national incidence of diabetes mellitus types 1 and 2. The ageing population and incidence of diabetes are projected to continue to rise, suggesting that, unless significant successful interventions are implemented, the incidence of NOE is also likely to continue to rise. Antibiotic resistance does not appear to be a significant contributing factor.

It is a challenge to measure and correlate clinician awareness of NOE with its rising incidence, though this cannot be ruled out as a factor in the documented rise in reported cases of NOE nationally. It is important to remember that correlation does not imply causality. Increasing clinician awareness of NOE may have led to more diagnoses but is also likely to have improved the diagnosis and management of NOE, contributing to better patient outcomes.

While this study provides important insights, it has several limitations. The analysis relies exclusively on publicly accessible data. Examining trends at population level, it lacks patient-specific data and cannot establish direct causal relationships. Nonetheless, the findings point to several significant trends over the study period, offering a foundation for deeper exploration in future studies.

## Data Availability

Primary data are available as supplemental files.
